# Supplementary material for: Causal interplay between lactose intolerance and gut microbiota: a combined bidirectional Mendelian randomization and in vivo validation study
Source: Front Nutr. 2026 Jun 1;13:1803337. doi: 10.3389/fnut.2026.1803337 (PMC13265576; doi:10.3389/fnut.2026.1803337)
Supplement: Supplementary file 1 [file Data_Sheet_1.zip › supplementary materials/Forward/leave one out cross validation/ebi-a-GCST90027810.finngen_R12_E4_LACTONAS.pdf]

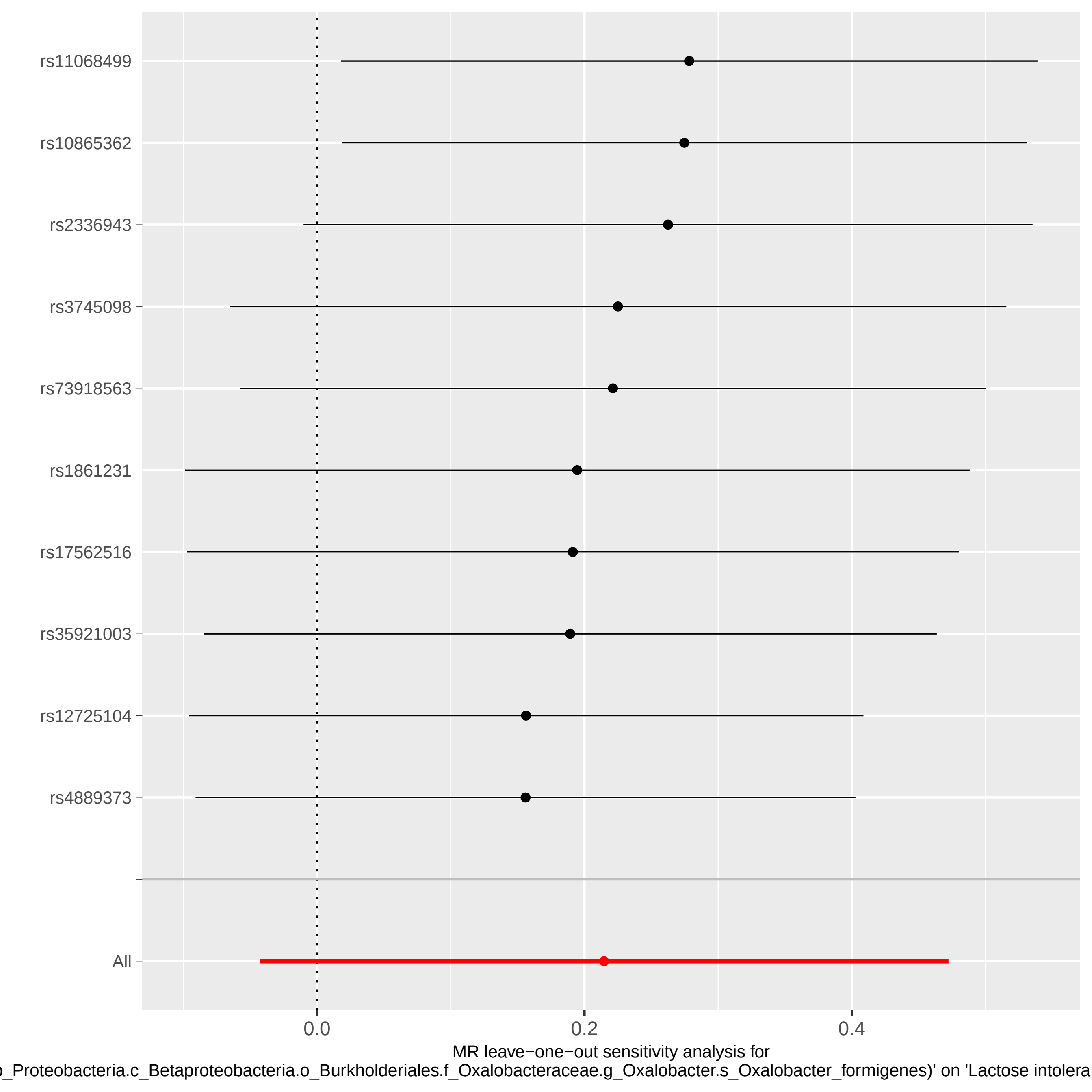

rs11068499
rs10865362
rs2336943
rs3745098
rs73918563
rs1861231
rs17562516
rs35921003
rs12725104
rs4889373
All
0.0
0.2
0.4
MR leave−one−out sensitivity analysis for
o_Proteobacteria.c_Betaproteobacteria.o_Burkholderiales.f_Oxalobacteraceae.g_Oxalobacter.s_Oxalobacter_formigenes)' on 'Lactose intolera
